# Uncovering key predictive channels and clinical variables in the gamma band auditory steady-state response in early-stage psychosis: a longitudinal study

**DOI:** 10.1017/neu.2024.60

**Published:** 2024-12-09

**Authors:** Kristina M. Holton, Amy Higgins, Austin J. Brockmeier, Mei-Hua Hall

**Affiliations:** 1 Center for Bioinformatics and Computational Biology, University of Delaware, Newark, DE, USA; 2 Psychosis Neurobiology Laboratory, McLean Hospital, Belmont, MA, USA; 3 Department of Electrical and Computer Engineering, University of Delaware, Newark, DE, USA; 4 Department of Computer and Information Sciences, University of Delaware, Newark, DE, USA; 5 Department of Psychiatry, Harvard Medical School, Boston, MA, USA; 6 Division of Psychotic Disorders, McLean Hospital, Belmont, MA, USA

**Keywords:** Early stage psychosis, electroencephalogram, machine learning, longitudinal, schizophrenia spectrum disorder

## Abstract

**Objective:**

Psychotic disorders are characterised by abnormalities in the synchronisation of neuronal responses. A 40 Hz gamma band deficit during auditory steady-state response (ASSR) measured by electroencephalogram (EEG) is a robust observation in psychosis and is associated with symptoms and functional deficits. However, the majority of ASSR studies focus on specific electrode sites, while whole scalp analysis using all channels, and the association with clinical symptoms, are rare.

**Methods:**

In this study, we use whole-scalp 40 Hz ASSR EEG measurements – power and phase-locking factor – to establish deficits in early-stage psychosis (ESP) subjects, classify ESP status using an ensemble of machine learning techniques, identify correlates with principal components obtained from clinical/demographic/functioning variables, and correlate functional outcome after a short-term follow-up.

**Results:**

We identified significant spatially-distributed group level differences for power and phase locking. The performance of different machine learning techniques and interpretation of the extracted feature importance indicate that phase locking has a more predictive and parsimonious pattern than power. Phase locking is also associated with principal components composed of measures of cognitive processes. Short-term functional outcome is associated with baseline 40 Hz ASSR signals from the FCz and other channels in both phase locking and power.

**Conclusion:**

This whole-scalp EEG study provides additional evidence to link deficits in 40 Hz ASSRs with cognition and functioning in ESP, and corroborates with prior studies of phase locking from a subset of EEG channels. Confirming 40 Hz ASSR deficits serves as a candidate phenotype to identify circuit dysfunctions and a biomarker for clinical outcomes in psychosis.

## Graphical abstract

**Figure:**
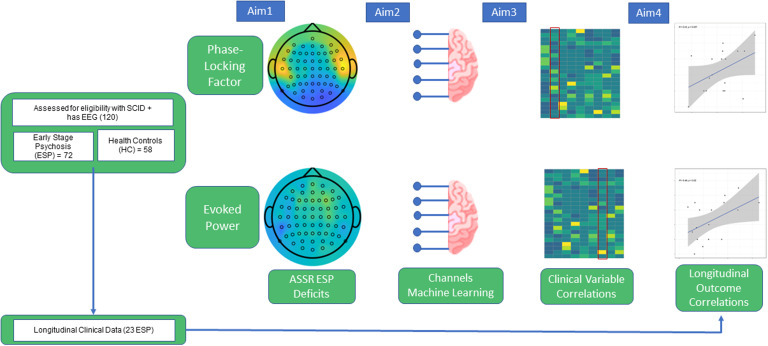

Significant outcomes

- Novel evidence of 40 Hz ASSR in ESP patients across whole-scalp EEG measures of phase locking and power.
- Patterns in whole-scalp 40 Hz ASSR EEG channel measurements, especially phase locking, identified by machine learning, and focused on a subset of key EEG channels that serve as candidate biomarkers for early-stage psychosis.
- Phase-locking factor is correlated with cognitive measures, and baseline 40 Hz ASSRs are correlated with short-term longitudinal functional outcomes.

Limitations

- Reduced statistical power in longitudinal analysis due to limited sample size caused by the COVID-19 pandemic.
- Analysis of early-stage psychosis case ignores the heterogeneity of SSD and AP diagnoses.
- Network-level and source-level disruption of gamma oscillations in ESP are not investigated in the study.

Highlights

- 40 Hz ASSR deficits are already present in ESP, and PLF outperforms PWE in predictive and discriminative power through machine learning, while focusing on a subset of channels.
- There is a strong correlation between cognitive measures and PLF.
- Baseline 40 Hz ASSR is correlated with longitudinal functioning at a short-term follow-up.

## Introduction

Psychotic disorders are characterised by aberrant sensory processing, cognitive deficits, and psychosocial functioning impairment. Within the sensory modality, the electroencephalogram (EEG) recording of auditory steady-state response (ASSR) reflects evoked oscillatory responses to modulated stimuli and induced oscillations peaking at 40 Hz to rapidly presented periodic auditory stimulation (Thune *et al*., 2016). For stimuli modulated at 40 Hz, ASSR is both evoked and induced. The 40 Hz ASSR is of particular importance because it lies in the range of gamma oscillations, which are robustly altered in major brain disorders, such as schizophrenia and psychosis (O’Donnell *et al*., 2013, Onitsuka *et al*., 2022). Disruptions in 40 Hz ASSR have been consistently observed in psychotic disorders including schizophrenia spectrum and affective psychosis as well as linked to cognitive function and clinical symptoms in these disorders, and are also considered as a potential biomarker for these conditions (Spencer *et al*., 2008b; Uhlhaas and Singer, 2010; Mulert *et al*., 2011; Thune *et al*., 2016; Zhou *et al*., 2018; Onitsuka *et al*., 2022).

ASSR in the cerebral cortex are primarily generated by the reciprocal interactions between excitatory pyramidal cells and parvalbumin-expressing (PV+) inhibitory interneurons (basket cells) in local circuits (Buzsaki and Wang, 2012). It is proposed that the imbalance of inhibitory and excitatory (E/I) neural circuits is an underlying mechanism for psychosis including schizophrenia spectrum disorders (SSD) (Hirano and Uhlhaas, 2021; Onitsuka *et al*., 2022) and bipolar disorder with psychosis and major depressive disorder with psychotic features, also known as affective psychosis (AP) (Spencer *et al*., 2008a; Hall *et al*., 2012; Johannesen *et al*., 2013). The imbalances of E/I circuits are already detectable in early-stage psychosis (ESP), as evidenced by deficits compared to healthy controls in the 40 Hz ASSR (Spencer *et al*., 2008b, Grent-’t- Jong *et al*., 2021).

Although the 40 Hz ASSR is primarily generated in the primary auditory areas, other brain regions, including the frontal lobe (Koshiyama *et al*., 2020; Tada *et al*., 2021; Koshiyama *et al*., 2021a), thalamus (Steinmann and Gutschalk, 2011; Grent-’t-Jong *et al*., 2021), hippocampus (Grent-’t-Jong *et al*., 2021), and parietal cortex (Tada *et al*., 2021) also contribute to ASSR generation. Koshiyama et al. show that in patients with schizophrenia, localised deficits of the 40 Hz ASSR in the auditory cortex quickly propagate to other brain regions, especially the frontal lobe, suggesting a network-level disruption of gamma oscillations (Koshiyama *et al*., 2021a). The inability of the frontal regions to engage properly is associated with wider cognitive and functional deficits.

Cognitive dysfunction and social occupational function impairment are core features of psychotic disorders (Addington and Addington, 2000; Kahn and Keefe, 2013; Kalin *et al*., 2015; Koshiyama *et al*., 2021b). To assess impairments in cognitive processes, the Matrix Consensus Cognitive Battery (MCCB), a standardised assessment tool, evaluates various cognitive domains affected by psychosis, including working memory, processing speed, attention, problem solving, verbal and visual learning, and social cognition (August *et al*., 2012). To measure functioning, the Global Assessment of Functioning (GAF) (Endicott *et al*., 1976) is widely used for assessing overall functional level, while the modified Multnomah Community Ability Scale (MCAS) is used for assessing day-to-day activities and social occupational functions (Hendryx *et al*., 2001; Chan *et al*., 2021). In addition to these deficits, the severity of symptoms, both psychosis and mood, may fluctuate over time and can be measured through the Positive and Negative Symptoms Scale (PANSS) (Kay *et al*., 1987), the Montgomery and Asberg Depression Rating Scale (MADRS) (Montgomery and Asberg, 1979), and Young’s Mania Rating Scale (YMRS) (Young *et al*., 1978).

EEG recordings of ASSRs are quantified by two measurements: evoked power or power of waveform envelope (PWE) and inter-trial phase-coherence, which we refer to herein as phase-locking factor (PLF). These measurements are computed from the EEG recordings using time-frequency analysis methods, specifically the wavelet transformation. Evoked power measures the overall strength or amplitude of the oscillations in response to the auditory stimuli. Relatively higher evoked power means that a larger population of neurons are synchronously firing in response to the auditory stimuli (Spencer *et al*., 2008a). PLF measures the consistency in the phase of the oscillations across different trials. If the phase of the oscillations is consistently aligned to the onset of the auditory stimuli across different trials, this results in a high phase-locking factor (Spencer *et al*., 2008b).

While 40 Hz ASSR deficits have been consistently observed in SSD and AP patients, studies have typically focused on a few channels on the frontal central sites of the EEG (Onitsuka *et al*., 2022). Analysing ASSR activity from the whole-scalp EEG channels offers an advantage over using a few EEG channels by providing a more comprehensive and accurate representation of brain activity, leading to more reliable and clinically relevant insights. Whole-scalp analysis captures the full spatial distribution of ASSR responses, enhancing the ability to detect subtle abnormalities and differentiate between localised versus widespread dysfunction. This approach reduces the risk of missing important neural signals that might be impaired in patients but undetected by a limited number of channels, offering a richer and more detailed picture of neural activity. While source-level analyses are valuable for pinpointing the spatial origin of ASSR deficits (Grent-’t-Jong *et al*., 2021; Koshiyama *et al*., 2021b), scalp-level ASSR provide clinically meaningful information despite being more global due to volume conductance (Nunez and Srinivasan, 2006). It reflects the overall integrity of the auditory system, including how well the brain can synchronise with external auditory stimuli. In psychosis, disruptions in these processes are often detectable at the scalp level and are associated with broader cognitive and functional impairments. Thus, scalp-level ASSR can serve as a biomarker for identifying patients at risk of poorer outcomes, guiding interventions, and tailoring treatment plans (Javitt *et al*., 2008; Donde *et al*., 2023).

Machine learning applied to these EEG data might provide a means to accurately predict disease status and trajectories (Barros *et al*., 2021), complementing clinical variables with EEG-based biomarkers. Machine learning methods can handle multivariate and complex data and extract or select representations of relevant data features for training classifiers (Saeidi *et al*., 2021). The accuracy of the classifier depends on the choice of the classifier algorithm or functional form. Administering a battery of classifiers in both PLF and PWE can reveal which type of classifier performs the best with respect to each paradigm, while permutation analysis can establish the robustness of the classifiers. With some types of classifiers, the importance of each channel in either PLF or PWE can be assessed directly, but for other classifiers the importance is assessed by examining the classification accuracy with and without the channel. Taking this idea further, one can create ensemble rankings of each channel for PLF and PWE across the different classifiers revealing highly discriminatory channels. Through this machine learning approach, we assess EEG patterns in whole-scalp 40 Hz ASSR as potential biomarkers of psychosis. To date we are unaware of studies of ASSR applying machine learning methods on EEG with whole-scalp electrodes, and therefore, this study focusses on addressing this gap.

Additionally, studies have found associations that ASSR PLF or PWE deficits in psychosis are associated with clinical symptoms and functioning in patients (Mulert *et al*., 2011; Zhou *et al*., 2018; Koshiyama *et al*., 2021b). However, testing clinical assessments individually is statistically suboptimal, given that many of these assessments are interrelated, either positively or negatively. Using principal component analysis (PCA) to group correlated clinical variables into components improves our ability to understand and interpret the relationships between clinical variables and channel-wise measurements of PLF and PWE.

Further, there is an urgent need to accurately characterise the progression of symptoms and understand the neurophysiological changes during the ESP period because early detection and intervention may likely slow functional decline and promote favourable outcomes (McGorry, 2002; Pantelis *et al*., 2005). Since the majority of studies of gamma oscillations report chronic schizophrenia patients (Thune *et al*., 2016), the potential to assess longitudinal functional outcomes in ESP subjects using 40 Hz ASSR is only recently being realised. Koshiyama and colleagues found that 40 Hz ASSR was significantly reduced in the recent onset schizophrenia and ultra-high-risk for psychosis groups and that the attenuated 40 Hz ASSR was correlated with future (12 month) global functioning level (GAF) in the schizophrenia group (Koshiyama *et al*., 2018). However, this study only reported correlations for a single electrode site.

In this study, we apply machine learning and data science approaches to study whole-scalp 40 Hz ASSR in ESP patients. The dataset consists of a longitudinal cohort (both SSD and AP, *N* = 72) and age matched healthy controls (*N* = 58). We propose to **i)** examine whether 40 Hz ASSR PLF and PWE deficits are already present at early stage of illness, aligning with san earlier study (Spencer *et al*., 2008b); **ii)** apply machine learning to construct classifiers using whole-scalp PLF and PWE channels to examine which channels are the most important to discriminate between ESP and HC and what type of classifier is optimal; **iii)** correlate PLF and PWE channels with the principal components that underlie clinical variables, in order to identify which components and channels are correlated; and **iv)** examine correlations between baseline PLF and PWE with clinical variables at one-year follow-up to gain predictive insights into short-term functional outcome. Our study explores different machine learning techniques and utilises either PLF or PWE to not only capture differences between ESP and HC, but also discover the channels most indicative of deficits and relevant to clinical variables. The results provide a framework for better understanding 40 Hz ASSR measurements as biomarkers.

## Methods

### Study sample

**Figure 1.**
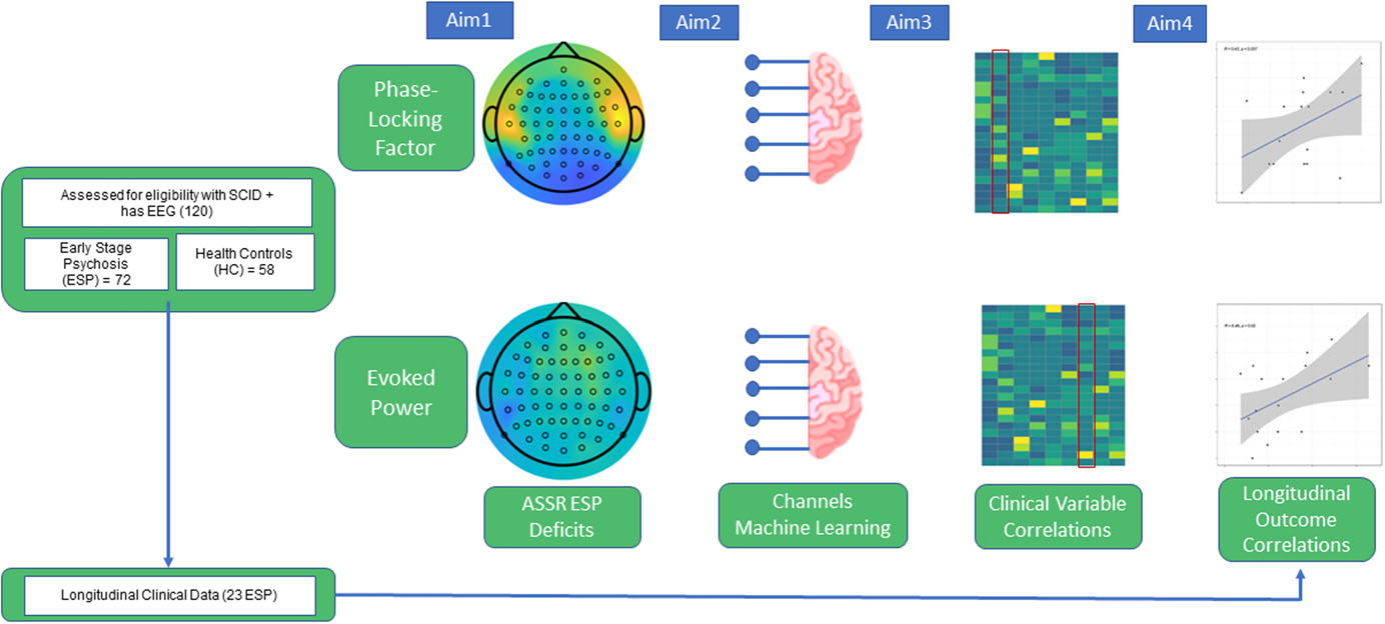
Study schematics.

Subjects consisted of 130 participants, with a total of 29 schizophrenia spectrum disorder (SSD), 43 affective psychosis (AP), and 58 healthy controls (HC), under the approval of the McLean Hospital Institutional Review Board. The study schematic is shown in Fig. 1, and demographics are provided as Supplementary Table 1.

Characteristics of this study cohort have been described in (Chan *et al*., 2020). Briefly, for inclusion criteria, patients were recruited from the First Episode Psychosis Clinic (McLean OnTrackTM) at McLean Hospital (Belmont, MA) and met DSM-IV criteria for Schizophrenia, Schizoaffective disorder, Bipolar I Disorder with psychotic features, or psychosis not otherwise specified, as assessed by the Structured Clinical Interview for DSM-IV (SCID) and review of medical records at their initial assessment. ESP at the time of entry to the clinic was defined as having an onset of psychotic symptoms within the past six years, and was not based on hospital admission or date or clinical intervention. Age-matched healthy controls (HCs) were recruited from the community and evaluated by SCID-NP. Exclusion criteria for ESP participants included psychotic symptoms that were attributable to acute intoxication or drug use, and illness duration over six years. Exclusion criteria of all subjects included: diagnosis of neurological disorders; history of head trauma with loss of consciousness; hearing impairments, blindness, or deafness; electroconvulsive therapy within the past 6 months; and IQ less than 70. Additional exclusion criteria for HCs were the following: no current or past history of psychotic or affective disorders, no current substance abuse or lifetime substance dependence, and no first-degree relative with a history of psychosis or bipolar disorder.

### Clinical assessments

Clinical measures used in this study included: the Positive and Negative Syndrome Scale (PANSS) with subscales for Positive, Negative, and General symptoms (Kay *et al*., 1987), the MADRS (Montgomery and Asberg, 1979), Young Mania Rating Scale (YMRS) (Young *et al*., 1978), a modified Multnomah Community Ability Scale (MCAS) (Hendryx *et al*., 2001; Chan *et al*., 2021), and the GAF (Endicott *et al*., 1976). Cognition domains were measured using the MCCB (August *et al*., 2012). Medication information, including antipsychotics and lithium dosage, was collected at each assessment timepoint. Antipsychotics (96% second-generation) were converted into chlorpromazine equivalents based on the recommendations of Gardner et al., (Gardner *et al*., 2010).

### Electrophysiological recording and processing

The electroencephalogram (EEG) was recorded using the BioSemi Active Two system (BioSemi Inc., Amsterdam, Netherlands) with a bandpass of DC–104 Hz at a sampling frequency of 512 Hz, and a Common Mode Sense as the reference (PO2 site) using either an 18- or 64-channel electrode cap. Electrooculogram (EOG) electrodes were placed below and at the outer canthi of the left eye. Fifteen subjects were recorded using an 18-channel BioSemi Active Two system. Their PLF and PWE values were imputed to 59 channels (PLF) and 63 channels (PWE) using a K-nearest neighbours approach, 10 neighbours, with optimal impute (iai v1.5.0).

The 40 Hz ASSR stimuli were presented through earphones in one block of stimuli (150/block) (Zhou *et al*., 2018). Stimuli consisted of trains of 1-ms white noise clicks (500-ms duration, 1100-ms stimulus onset asynchrony, 80-dB sound pressure level). Subjects were instructed to look at the fixation cross on the monitor and listen to the stimuli.

Signal processing was performed off-line using Brain Vision Analyzer software (Brain Products GmbH, Germany) and blind to group membership (Kozhemiako *et al*., 2022). EEG data were downsampled to 256 Hz, re-referenced off-line to the linked mastoids, and filtered with a passband between 0.1 and 50 Hz. Single trial segments were extracted, baseline-corrected relative to the 500 ms pre-stimulus interval, eye-blink-corrected using Brain Vision Analyzer’s default setting (Gratton *et al*., 1983) and artefact rejected if values exceeded>100 μV. Phase locking (PLF) and evoked power (PWE) at each site were calculated on wavelet coefficients obtained from Morlet wavelet transformation of the segmented data (representing the 1–50 Hz frequency range, with a total number of 50 frequency layers using a Morlet parameter of 10). The PLF quantifies consistency of oscillatory phase across individual trials, ranging from 0 (purely non-phase-locked activity) to 1 (fully phase-locked activity). Gamma-band (40 Hz) PLF and PWE were computed by averaging across the 36–46 Hz wavelet frequency layers in the 20–520 ms window post wherein both PLF and PWE were maximal for 40 Hz ASSR (Kozhemiako *et al*., 2022). Averaged HC and ESP responses as time-frequency spectrograms for PLF and PWE were created from representative channels across the scalp using Brain Vision Analyzer software.

### Assessing case-control differences

PLF and PWE channels were individually assessed for differences between ESP and HC using a one-sided (alternative=’greater’ for ESP compared to controls) using a Welch’s *t*-test (R version 4.1.2). Inverse log10 *p*-values were plotted on scalp maps using MATLAB script plot.topography.m (version 1.5) (Martínez-Cagigal, 2020) as scaled to the most extreme p-value (0.00138).

### Modeling PLF and PWE channels with machine learning

PLF and PWE channels were used to predict ESP/HC by splitting subjects 80:20 into train and test sets stratified by case. Model training and hyperparameter selection was performed on the train set (80% of the subjects) and tested on the 26 held-out test subjects. Hyperparameter choices over a grid search of 100 bootstraps and final selection are reported in Supplementary Table 2. Random forest used 100 out-of-bag times, with mean Gini index reported (randomForest v4.7–1.1 (Liaw and Wiener, 2002), caret v6.0–90 (Kuhn and Max, 2008)). Ridge (L2-penalized elastic net regression) underwent 100 bootstraps with importance reported (glmnet v4.1–4 (Friedman *et al*., 2010; Tay *et al*., 2023), caret v6.0–90 (Kuhn and Max, 2008)). Gaussian process with a radial basis function (RBF) underwent 100 bootstraps, with feature importance reported as a measure of area under the curve (AUC) of the receiver operator characteristic curve (ROC) for each feature (kernlab v0.9–31 (Karatzoglou *et al*., 2004; Karatzoglou *et al*., 2023), caret v6.0–90 (Kuhn and Max, 2008)). Support vector machine (SVM) with a RBF kernel underwent 100 bootstraps, with importance reported as a measure of AUC of the ROC for each feature (kernlab v0.9–31 (Karatzoglou *et al*., 2004; Karatzoglou *et al*., 2023), caret v6.0–90 (Kuhn and Max, 2008)). Naive Bayes underwent 100 bootstraps, with feature importance reported as a measure of AUC of the ROC for each feature (naivebayes v0.9.7 (Majka, 2024), caret v6.0–90 (Kuhn and Max, 2008)). For all algorithms, we calculated the test set F1, accuracy, accuracy upper/lower 95% confidence intervals, balanced accuracy, AUC, sensitivity, specificity, positive predictive value, negative predictive value, and root mean standard error (RMSE) (caret v6.0–90 (Kuhn and Max, 2008), ModelMetrics v1.2.2.2 (Hunt, 2020)).

We created an ensemble ranking of features across the ESP-HC deficits *t*-test and machine learning algorithms. Importance of clinical features from each algorithm was given an ordinal rank, and to ensemble, ranks were averaged across algorithms and sorted, assigning a new ordinal rank according to sorting. Plots were created with ggplot2 (v3.3.5) and RColorBrewer (v1.1–2).

To test the importance of correct feature labelling to the classifiers, we randomly scrambled the channel labels of the PLF and PWE data matrices 20 times, and calculated the test set performance measures’ means and standard deviations across the trials. Original performance and scrambled F1 performance were demonstrated via boxplot, created with ggplot2 (v3.3.5) and RColorBrewer (v1.1–2).

### Assessing clinical variables correlated with channels’ responses

We decomposed the clinical variables into principal components. We first selected the subset of clinical variables with 80% complete data, then took only ESP patients with complete data across these features, which yielded 24 clinical variables and 46 ESP patients. We assessed the correlation of clinical variables via Pearson correlation and generated a corrplot (ggcorrplot 0.1.4). We performed PCA decomposition of the clinical variables, and evaluated the eigenvectors, retaining 9 principal components (PCs). The clinical variables driving each PC were thresholded by>10% contribution (factoextra 1.0.7). Each EEG channel’s PLF and PWE measurements were correlated with each of the coordinates for the 9 PCs using a Pearson correlation test, two-sided. P-values were corrected for multiple testing using Benjamini-Hochberg false discovery rate (FDR). The contribution of the clinical variables to the principal components were plotted via heatmap (pheatmap 1.0.12).

### Assessing baseline PLF / PWE with clinical variables at baseline and one-year follow-up

Baseline and one-year follow-up clinical measures were correlated for the clinical assessments from 23 patients (8 SSD, 15 AP) via Pearson correlation, two-sided alternative hypothesis. Baseline PLF and PWE channels were correlated with baseline clinical measures and one-year follow-up clinical assessments from 23 patients (8 SSD, 15 AP) via Pearson correlation, one-sided alternative hypothesis as indicated per variable. Functioning (GAF, MCAS) range from high (less deficit) to low (more deficit) and have the alternative hypothesis of ‘greater’, whereas disease severity as measured by PANSS (all), YMRS, MADRS range from low (less severe) to high (most severe) and have the alternative hypothesis of ‘less’. (Supplementary Table 9, Supplementary Table 10). Correlation linear model plots were created with ggplot2 (v3.3.5) and ggpubr (v0.4.0).

## Results

### Comparing case-control ASSR

**Figure 2.**
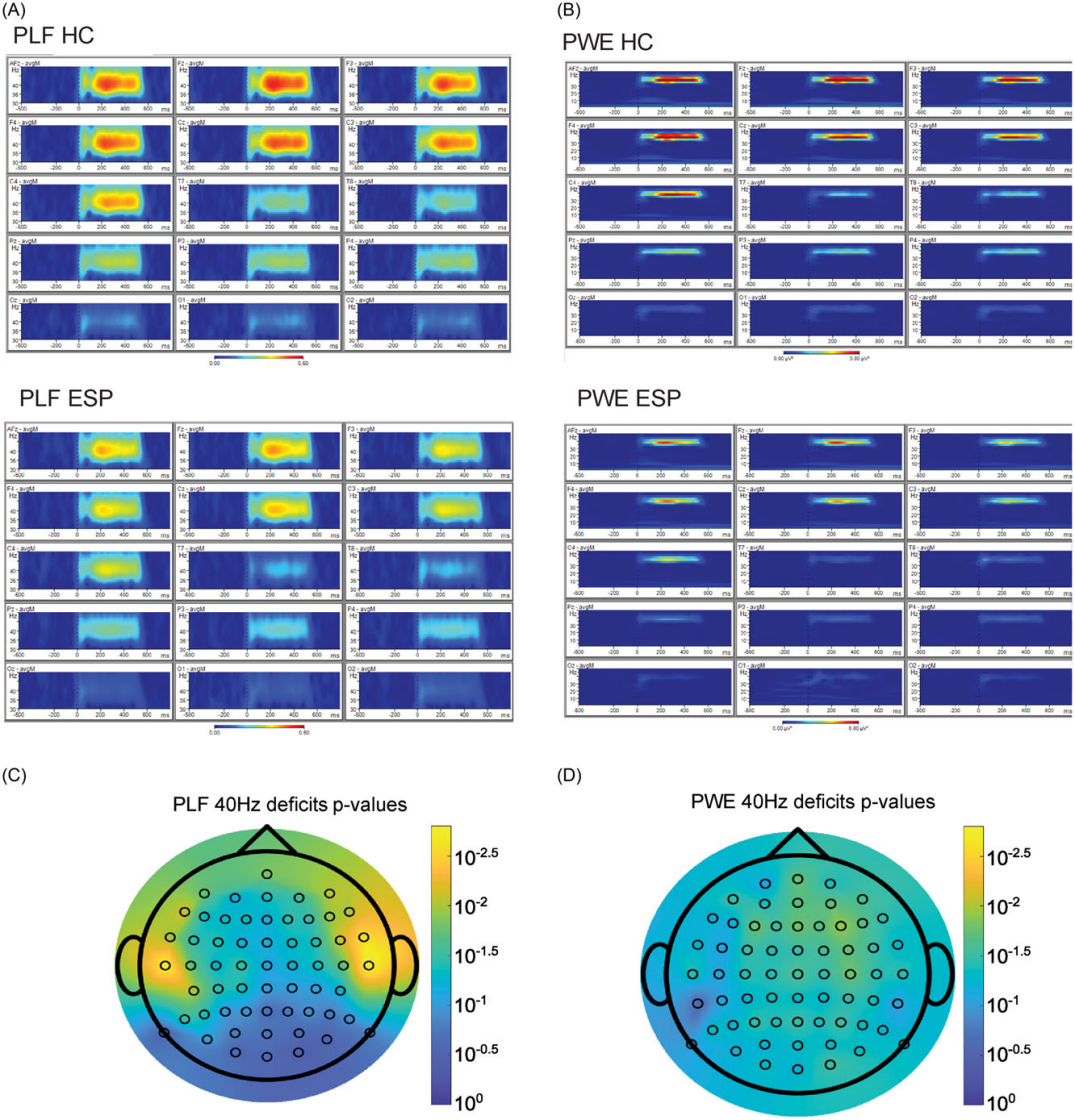
**ESP deficits across channels in PLF and PWE.** Average 40 Hz ASSR frequency spectrograms over representative channels for HC (*N* = 58; top) and ESP (*N* = 72; bottom) for PLF (**A**) and PWE (**B**). Channels from left-right are AFz, Fz, F3, F4, Cz, C3, C4, T7, T8, Pz, P3, P4, Oz, O1, O2. For each channel in PLF (**C**) and PWE (**D**), a one-sided student’s t-test with the alternative hypothesis of ‘greater’ was run for HC versus ESP. Scale is inverse log10 p-value, with a maximum p-value of 0 and a minimum p-value of 0.00138.

We compared the gamma band (40 Hz) ASSR response in HC versus ESP in both the PLF and PWE paradigms, demonstrated visually through representative channels as frequency spectrograms (Fig. 2A–B). We assessed these differences via a one-sided *t*-test, and found that deficits already exist at ESP (Fig. 2C–D). In PLF, a pattern of fronto-parietal deficits are present, but with the smallest *p*-values in the temporal T8 (*p* = 0.00137) and T7 (*p* = 0.0015) electrodes. PLF had 30 significant channels at a significance threshold (alpha) of *p* ≤ 0.05. In PWE, the pattern of deficits is centred around Fz and the frontal lobe, with 46 significant channels at a significance threshold (alpha) of *p* ≤ 0.05. The minimal *p*-value in PLF is 0.00138 for channel T8 whereas the minimal *p*-value for PWE is 0.0111 for F4. The full list of *p*-values are available in s = Supplementary Table 3.

### Machine learning models classify case/control status from channels

We evaluated multiple machine learning techniques – random forest (RF), elastic net linear model with L2 penalisation (ridge), Gaussian process with a radial basis function (Gaussian radial), SVM with a radial basis kernel (SVM radial), and naive Bayes – to classify whether a participant was case or control using the PLF or PWE channels.

**Figure 3.**
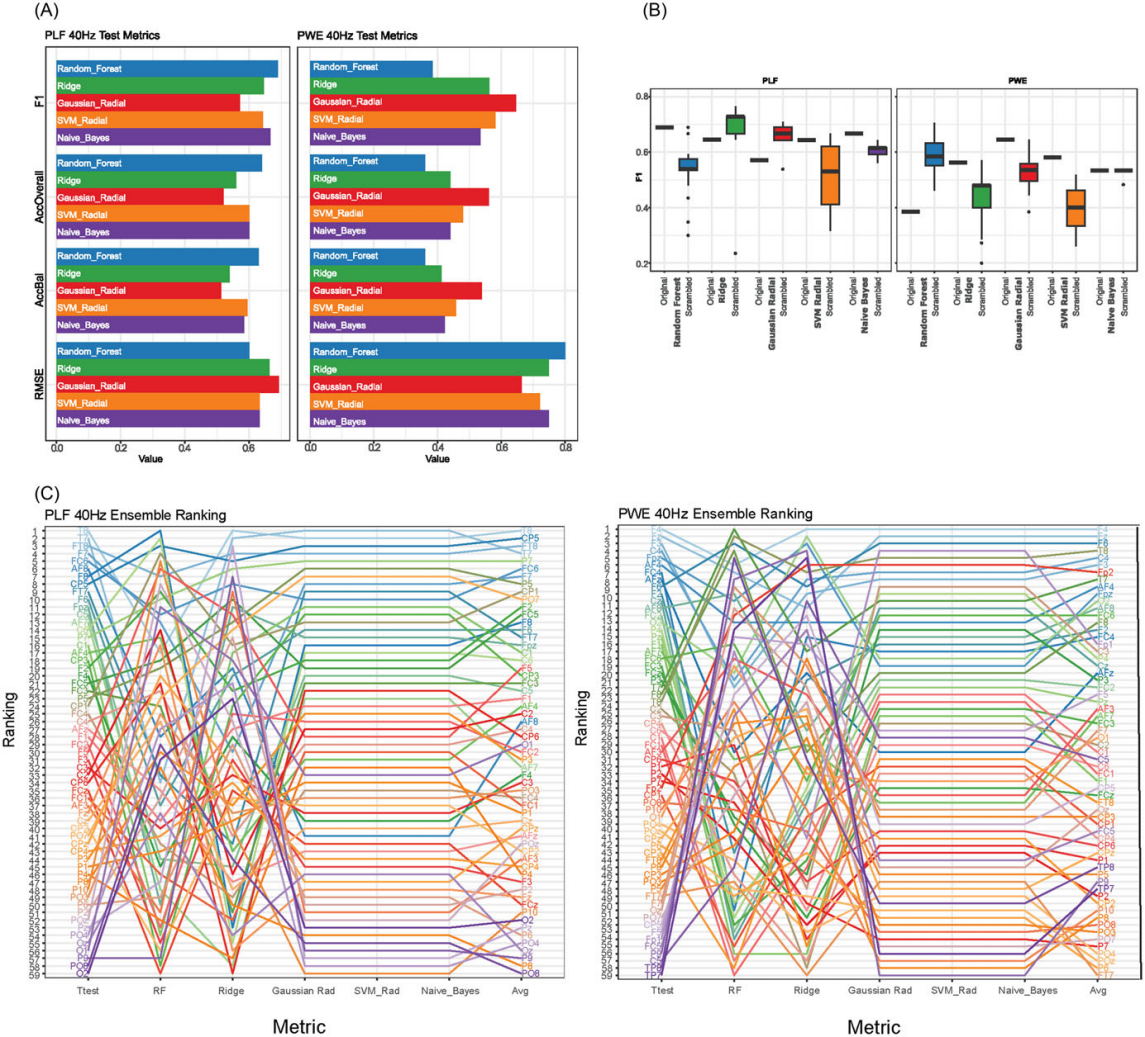
**Machine learning metrics for PLF and PWE. (A)** For PLF and PWE test, machine learning metrics F1, overall accuracy (AccOverall), balanced accuracy (AccBal), and root mean square error (RMSE) are displayed for random forest (RF), Ridge (L2 elasticnet), Gaussian process with a radial kernel (Gaussian Radial), support vector machines with a radial kernel (SVM Radial), and naive Bayes. **(B).** For PLF and PWE, original test F1, and scrambled labels F1 over 20 permutations for each machine learning algorithm are demonstrated via boxplot. **(C)** For PLF and PWE, ensemble ranking metrics for *t*-test (increasing p-value), RF (mean Gini index), Ridge (beta coefficients), Gaussian Radial (AUC), SVM radial (AUC), Naive Bayes (AUC), and average rank across all six metrics (avg_rank) are displayed.

In the PLF paradigm, RF had the best F1 (0.69), accuracy (0.64), and balanced accuracy (0.63), and lowest root mean square error (RMSE) (0.60) in the test data. Naive Bayes had the second highest F1 (0.67) and performed similarly to RF. The Gaussian radial had the lowest F1 (0.57), accuracy (0.52), and balanced accuracy (0.51), with the highest RMSE (0.69) (Fig. 3A, Supplementary Table 4). The chance level of accuracy is 0.55 given 72 cases and 58 healthy controls.

In contrast, the PWE paradigm had markedly less classification power and the performance across techniques varied markedly from the PLF paradigm. RF performed the worst with less than chance performance in F1 (0.38), accuracy (0.36), and balanced accuracy (0.36), and had the highest RMSE (0.80). Of the five algorithms, Gaussian radial performed the best, in F1 (0.65), accuracy (0.56), balanced accuracy (0.54), and had the lowest RMSE (0.66) (Fig. 3A Supplementary Table 4).

To better understand the importance of feature specificity for these techniques, we scrambled the feature labels 20 times and assessed F1, accuracy, and AUC. Scrambling the feature labels caused RF’s performance to drop off in PLF, suggesting its specificity in channel selection, whereas RF’s performance improved in PWE, which was previously below chance—indicating RF was overfitting in PWE. Performance for Gaussian radial markedly decreased in PWE indicating there existence of meaningful pattern across the channels that is lost when scrambling (Fig. 3B, Supplementary Table 4).

For each machine learning algorithm, the importance of the features to the model was extracted. RF utilises mean Gini index, ridge is directly interpretable in terms of the elastic net beta coefficients, while Gaussian radial, SVM radial, and naive Bayes all use the area under the curve (AUC) of the receiver operator characteristic (ROC) for each independent channel. In particular, for the PLF RF algorithm, the five highest-important channels were F8, C6, CP5, CP1, and P10 (Supplementary Table 5).

We combined these rankings with the *t*-test rankings, and derived an ensemble (average) rank for each channel in both PLF and PWE. For PLF, channels T8, CP5, FT8, T7, and P7 were the five highest-ranked channels in their ability to discriminate between ESP and HC (Fig. 3C, Supplementary Table 5). Conversely, the five PWE channels best able to discriminate between ESP and HC were F4, Fz, F6, T8, and C4 (Fig. 3C, Supplementary Table 5).

### Combinations of clinical variables correlate with channels’ responses

**Figure 4.**
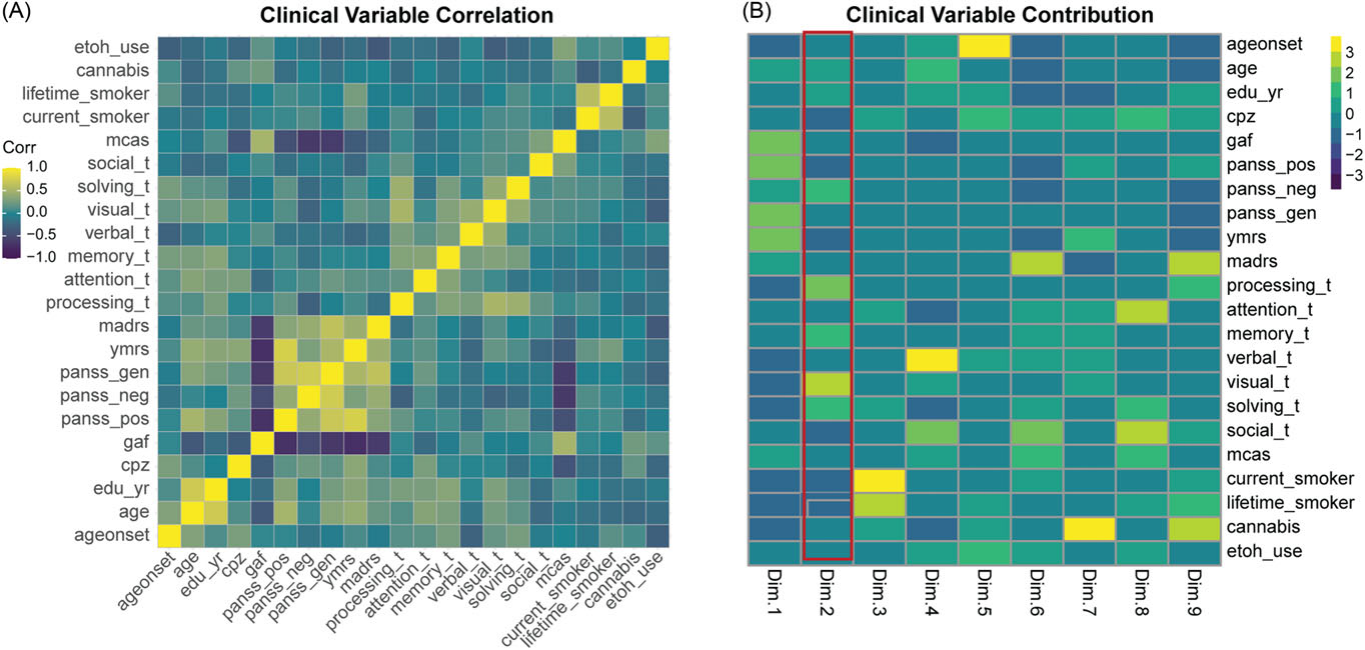
Clinical variables decomposed into principal components, and correlations to PLF and PWE. (A) Clinical variables with<20% missingness are correlated across 43 ESP with complete data via Pearson correlation. The Viridis colour scale shows high Pearson correlation value (yellow) to low Pearson correlation value (purple). **(B).** For the first 9 principal components, the contribution of each clinical variable is shown via colour bar, scaled per principal component. PLF demonstrates 52 channels had a significant Pearson correlation to PC2 (significance level of FDR ≤ 0.01) (highlighted in red).

For a subset of ESP with at least 80% completed clinical data (*N* = 46), we assessed the degree to which clinical variables are correlated, to find strong (absolute(R)>0.5) correlations between the PANSS scores, GAF and MCAS; MCCB subscores; and cannabis use (Fig. 4A, Supplementary Table 6). We performed PCA to decompose the clinical variables into components of highly-correlated features, thresholded by having>10% contribution to the principal component (PC). For the 46 ESP with complete data, we correlated each channel in PLF and PWE with the first nine PCs, which collectively explain 81% of the variance. The first PC has major contributions from GAF, PANSS Positive, PANSS Negative, and YMRS (Fig. 4B, Supplementary Table 6).

Across the components, PC2 and PLF measures on 42 channels had a Pearson correlation coefficient (R) greater than 0.3, with 52 channels that were significantly correlated (FDR ≤ 0.01). PC2 is represented by MCCB cognition measures (processing, memory, visual solving). (Fig. 4B, Supplementary Table 6, Supplementary Table 7). In contrast, we did not find strong correlation within clinical components for PWE measures at FDR<0.01 (Supplementary Table 6, Supplementary Table 8).

**Figure 5.**
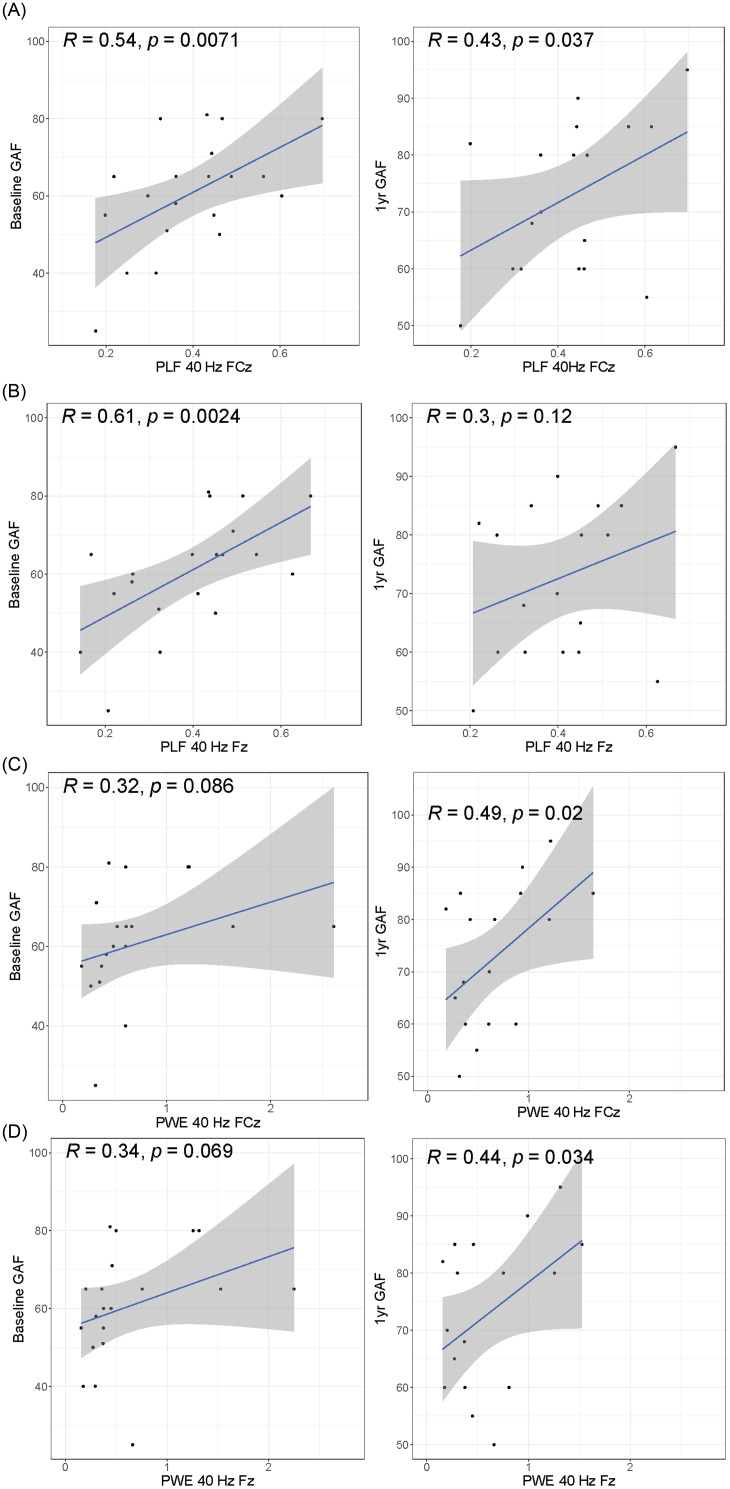
Correlations of baseline FCz and Fz to longitudinal GAF. For PLF and PWE (**A,C**), one-sided Pearson correlation (R) of baseline FCz to baseline and one-year GAF score are displayed, along with correlation test p-value. FCz is the *x*-axis, GAF score is the *y*-axis. Blue line is the linear model trend line, grey is the standard error. For PLF and PWE (**B,D**), one-sided Pearson correlation (R) of baseline Fz to baseline and one-year GAF score are displayed, along with correlation test p-value. Fz is the *x*-axis, GAF score is the *y*-axis. Blue line is the linear model trend line, grey is the standard error.

We further investigated the relationships between the ASSR and the individual clinical measures at baseline for the subset of patients (*N* = 23) with longitudinal data: baseline PLF correlated to baseline GAF in 22 channels, MCAS in 3, YMRS in 24, MADRS in 17, PANSS General in 15, PANSS Negative in 10, and PANSS Total in 22 channels. Baseline PWE did not correlate with baseline GAF in any channels at an alpha ≤ 0.05, but correlated to baseline MADRS in 24 channels, and YMRS in Cz (Fig. 5, Supplementary Table 9, Supplementary Table 10).

### Baseline channels predict functional outcome at follow-up

One-year follow-up clinical measures for a subset of patients (*N* = 23) were available. First, we measured the correlation of each clinical measure at baseline to its value at one year. Significant correlations were found for GAF (*R* = 0.75, *p* = 1.35e-3), MCAS (*R* = 0.804, *p* = 1.02e-4), and MADRS (*R* = 0.617, *p* = 8.35e-3) (Supplementary Table 11). We then examined correlations between baseline central channels FCz and Fz with clinical variables at the one-year follow-up. GAF was found to be significant at an alpha ≤ 0.05 in PLF FCz (*R* = 0.43, *p* = 0.037), PWE FCz (*R* = 0.49, *p* = 0.020), and PWE Fz (*R* = 0.44, *p* = 0.034) ] via a Pearson one-sided (alternative=’greater’) correlation test, as shown in Fig. 5, Supplementary Table 9, and Supplementary Table 10. We extended this analysis to measure the correlation of baseline 40 Hz to one-year GAF on all channels. In total, PLF had 11 channels significant (*p* ≤ 0.05) for GAF, while PWE had 21 (Supplementary Table 9, Supplementary Table 10).

Additionally, one-year PANSS Positive significantly correlated with baseline PWE at FCz (*R* = -0.39, *p* = 0.043) and one-year PANSS Total with PWE at Fpz (*R* = -0.40, *p* = 0.038) (Supplementary Table 10), while one-year YMRS was significantly correlated with PLF at F7 (*R* = -0.39, *p* = 0.044) (Supplementary Table 9).

## Discussion

Our study employs a comprehensive, whole-scalp approach to examine 40 Hz ASSR in ESP and HC and explores various machine learning techniques to identify the channels most indicative of deficits and relevant to clinical variables. Our findings reveal that **i**) ASSR deficits are already present in ESP; **ii**) PLF outperforms PWE in predictive and discriminative power through machine learning, and focuses on a subset of channels; **iii**) there is a strong correlation between cognitive measures and PLF; and **iv**) baseline 40 Hz ASSR is correlated with longitudinal functioning at a short-term follow-up. PLF has a more predictive, parsimonious signature than PWE, with a subset of frontal and temporal channels particularly relevant in PLF, while front-central channels are disrupted in PWE.

### Gamma band deficits are present in ESP

We found reduced gamma band ASSRs in ESP patients across multiple channels. This result is consistent with previous most reports in first episode psychosis patient (Spencer *et al*., 2008b; Tada *et al*., 2016; Alegre *et al*., 2017; Koshiyama *et al*., 2018), including a study using MEG approach (Grent-’t-Jong *et al*., 2021). This reiterates the utility of using the 40 Hz ASSR in assessing patient deficits in ESP. Across the whole scalp, the biggest differences between patients and controls in PLF are electrodes in the temporal sites (T7 and T8), consistent with evidence indicating the primary auditory cortex and superior temporal cortex being one key generators of 40 Hz ASSR (Draganova *et al*., 2007).

### Predicting case/control status from channels

We applied multiple machine learning techniques using channels across the whole scalp to examine which channels are the most important in discriminating ESP/control status at baseline. The best performing classifier is a random forest (RF) on PLF, with measurements modestly predictive with an F1 score of 0.69. The channels with the highest feature importance were F8, C6, CP5, CP1, and P10. In relation to the temporal sites (T7 and T8), F8, C6, and P10 form a triangle around T8, and CP5 and CP1 extend from T7. This indicates that the RF is using the patterns across these channels to robustly distinguish on an individual level ESP and HC. The best performing classifier for PWE is a Gaussian process (GP) with a radial basis function that achieves an F1 score of 0.65. Notably, switching the type of classifier from the other dataset yields much lower performance (yielding F1 scores of 0.57 for GP on PLF and 0.35 for RF on PWE). While both algorithms can create nonlinear decision boundaries, the specificity of the two types of classifiers to data is explainable. Random forests choose a set of decision trees based on channel specificity and is precise in channel selection in PLF, while Gaussian processes with a radial kernel use patterns across all channels and therefore do better with PWE. To test the validity of our results, we found that scrambling the channel labelling impeded the random forest’s ability to make accurate predictions in PLF. The Gaussian process’ predictive accuracy dropped when scrambling feature labels for PWE, hinting at specific patterning across the channels. Overall, the moderate F1 score for the best model may be due to small sample sizes and inherited heterogeneity of the patient population. Future studies with a larger cohort size (N) may be better poised to build a multi-diagnosis classifier.

### Utility of clinical variables in predicting channels’ responses

Few studies use clinical variables to relate to the ASSR channels’ responses (Mulert *et al*., 2011; Zhou *et al*., 2018). In this study, we implemented a PCA strategy to group correlated clinical variables into components and examined the correlation patterns of these components with each electrode channel across the whole scalp in PLF and PWE. Not surprisingly, we found that symptom severity scales (PANSS General, Negative, Positive, YMRS, and MADRS) all positively correlated with one another and negatively correlated with global functioning (GAF) and community functioning (MCAS) (i.e., high symptom score, low functioning score and low symptom score, high functioning) (Fig. 4A). Cognitive variables (processing speed, attention, memory, visual, and problem solving) from MCCB measures were modestly correlated with one another, and comprised principal component 2 (PC2) (Fig. 4A–B). Applying the PCA embedding to PLF, we found PLF was correlated with PC2 (88% within PC/8% across PC of total channels FDR ≤ 0.01, R range between 0.30 and 0.40). This result provides supporting evidence that 40 Hz ASSR impairment relates to cognitive function and attention mechanism (Parciauskaite *et al*., 2021; Coffman *et al*., 2022). In contrast, we did not find strong evidence of PWE in relation to the clinical components, but a few channels correlated with the component (PC7) associated with manic symptoms and cannabis use.

### Baseline channels predict longitudinal functional outcome

One prior study by Koshiyama et al., found a correlation between 40 Hz ASSR at FCz and future global functional outcome (GAF) in ESP (Koshiyama *et al*., 2018). We show that, in addition to FCz as reported in Koshiyama’s study, 10 PLF and 20 PWE channels at baseline all correlated with GAF at one year, that is, greater ASSR impairments at baseline predict lower functioning one year later (Supplementary Table 9, Supplementary Table 10). Baseline GAF is also highly correlated to one-year GAF, as is MCAS and MADRS (Supplementary Table 11). Most clinical measures had baseline correlations to PLF (except PANSS Total), but the correlations drop out at one-year follow-up. Interestingly, both PLF and PWE demonstrated baseline correlation to MADRS (PLF 17 channels, PWE 24 channels), which is consistent with findings in Parker et al (Parker *et al*., 2019). Although both baseline MCAS and GAF are highly correlated with follow-up MCAS and GAF, baseline ASSR only predicts one-year GAF. No significant longitudinal correlations were found for the MCAS measure. GAF is rated as an overall impression of symptomatic and functioning performance whereas the modified MCAS scores several different axes of day-to-day functionality independently (e.g., independent living, meaningful activity, social activity and relationships, management of money) (Hendryx *et al*., 2001; Chan *et al*., 2021). It is plausible that 40 Hz ASSR may be more sensitive in capturing an individual’s overall symptom and occupational functional state and that a larger sample is needed to have sufficient power for partitioning the precise functionality trajectories.

### Overall PLF and PWE signature

Ding and Simon have demonstrated that phase synchronisation, as measured by PLF, is much more sensitive to stimulus-synchronized neural activity than power (Ding and Simon, 2013). Specifically, gamma-band phase locking is not sensitive to modulations in signal amplitude, or changes in amplitude across subjects caused by differences in electrode impedance, and, thus, is a more robust measure of the underlying neural synchrony than gamma-band power. In this study, our findings corroborate this as PLF produced more extreme p-values on a subset of channels, PLF outperformed PWE in classification accuracy/F1 score, and showed stronger correlations with clinical variables.

### Right hemispheric laterality of deficits

Interestingly, we observed that there appears to be hemispheric asymmetries, specifically in PLF, with more prominent contribution of 40 Hz ASSR in the right hemisphere. For example, the random forest trained to classify ESP relied on channels F8 and CP6. Ensemble rankings of PLF features also implicate F4, F6, T8, and C4 as the most important features driving classification of ESP versus HC. This is consistent with the literature, in which ASSR showing right hemispheric dominance (Grent-’t-Jong *et al*., 2021)

## Supporting information

Holton et al. supplementary material 1 Holton et al. supplementary material

Holton et al. supplementary material 2 Holton et al. supplementary material

Holton et al. supplementary material 3 Holton et al. supplementary material

Holton et al. supplementary material 4 Holton et al. supplementary material

Holton et al. supplementary material 5 Holton et al. supplementary material

Holton et al. supplementary material 6 Holton et al. supplementary material

Holton et al. supplementary material 7 Holton et al. supplementary material

Holton et al. supplementary material 8 Holton et al. supplementary material

Holton et al. supplementary material 9 Holton et al. supplementary material

Holton et al. supplementary material 10 Holton et al. supplementary material

Holton et al. supplementary material 11 Holton et al. supplementary material

## Supplementary material

The supplementary material for this article can be found at https://doi.org/10.1017/neu.2024.60.
