# Supplementary material for: Uncovering key predictive channels and clinical variables in the gamma band auditory steady-state response in early-stage psychosis: a longitudinal study
Source: Acta Neuropsychiatr. 2024 Dec 9;37:e1. doi: 10.1017/neu.2024.60 (PMC13130324; doi:10.1017/neu.2024.60)
Supplement: Holton et al. supplementary material 3 — Holton et al. supplementary material [file S0924270824000607sup003.docx]

|  | Baseline Controls Mean | Baseline Controls SD | ESP Controls Mean | ESP Controls SD | Longitudinal ESP Mean | Longitudinal ESP SD |
| --- | --- | --- | --- | --- | --- | --- |
| **Age** | 22.690 | 3.808 | 22.778 | 3.537 | 23.783 | 4.285 |
| Age of Onset |  |  | 22.648 | 9.866 | 21.522 | 4.209 |
| Sex (%Male) | 65.517 |  | 63.889 |  | 56.522 |  |
| **Handedness (%Right)** | 93.103 |  | 77.778 |  | 91.304 |  |
| **Race (%white)** | 70.690 |  | 72.222 |  | 78.261 |  |
| **Diagnosis (%Bipolar)** |  |  | 59.722 |  | 65.217 |  |
| **Cannabis_Lifteime(%)** |  |  | 37.500 |  | 13.043 |  |
| **MCAS** | 54.776 | 0.587 | 47.478 | 5.001 | 50.950 | 3.953 |
| PANSS General |  |  | 28.030 | 7.290 | 21.950 | 5.083 |
| PANSS Negative |  |  | 12.224 | 4.951 | 9.900 | 4.103 |
| PANSS Positive |  |  | 13.030 | 6.502 | 10.600 | 4.999 |
| MADRS |  |  | 13.061 | 9.089 | 5.550 | 6.549 |
| YMRS |  |  | 7.231 | 7.634 | 4.600 | 6.886 |
| GAF |  |  | 59.766 | 13.583 | 72.778 | 13.414 |
| CPZE |  |  | 214.544 | 243.087 | 163.912 | 127.049 |

**Supplementary Table 1: Study demographics**. Baseline (N=130) and Longitudinal (N=23) subjects are stratified by HC and ESP, with Mean and standard deviation (SD) reported for each characteristic, except where percent (%) is denoted.
