# Supplementary material for: Uncovering key predictive channels and clinical variables in the gamma band auditory steady-state response in early-stage psychosis: a longitudinal study
Source: Acta Neuropsychiatr. 2024 Dec 9;37:e1. doi: 10.1017/neu.2024.60 (PMC13130324; doi:10.1017/neu.2024.60)
Supplement: Holton et al. supplementary material 4 — Holton et al. supplementary material [file S0924270824000607sup004.docx]

| Metric | Hyperparameter Choices | PLF Hyperparameters | PWE Hyperparameters |
| --- | --- | --- | --- |
| Random Forest | mtry=1:15 | mtry=1 | mtry=14 |
| Ridge (L2 Elasticnet) | alpha=0 lambda=0.001 to 0.1 by 0.001 increments | alpha=0  lambda=0.095 | alpha=0  lamda=0.099 |
| Gaussian Process w/ Radial Basis Function | sigma=0, 0.1, 0.2, 1, 2 | sigma=0.2 | sigma=1 |
| Support Vector Machines with Radial Basis Function | sigma=0, 0.1, 0.2, 1, 1  C=0.25, 0.5, 1, 2, 4 | sigma=0.1  C=4 | sigma=0.1  C=4 |
| Naïve Bayes | kernel=True/False  laplace=0, 0.5, 1  adjust=0.75, 1, 1.25, 1.5 | kernel=True  laplace=0  adjust=1.5 | kernel=True  laplace=0  adjust=1 |

**Supplementary Table 2**: **Machine Learning Hyperparameter Selection.** Each algorithm underwent 100 bootsraps (100 oob for Random Forest) to select the hyperparameters from the listed choice. The final PLF and PWE hyperparameters are reported.
